# Mobile Phone Addiction, Perceived Stress, and Morningness–Eveningness Preference in Nursing Students: A Computer‐Simulated Network Analysis

**DOI:** 10.1155/jonm/1540766

**Published:** 2026-06-30

**Authors:** Hongman Li, Zhenrong Shen, Yingting Jiang, Ying Xiong, Yihao Zeng, Qihan Zhang, Qiaoling Chen, Jiagen Xiang, Zengjie Ye

**Affiliations:** ^1^ School of Nursing, Guangzhou University of Chinese Medicine, Guangzhou, Guangdong Province, China, gzucm.edu.cn; ^2^ School of Nursing, Guangzhou Medical University, Guangzhou, Guangdong Province, China, gzhmc.edu.cn; ^3^ Medical College of Acu-Moxi and Rehabilitation, Guangzhou University of Chinese Medicine, Guangzhou, Guangdong, China, gzucm.edu.cn

**Keywords:** computer-simulated intervention, mobile phone addiction, moderated network analysis, morningness–eveningness preference, network analysis, perceived stress

## Abstract

**Background:**

Nursing students with high levels of perceived stress may be vulnerable to problematic mobile phone use. Morningness–eveningness preference may shape the relationship between mobile phone addiction and perceived stress. Using network analysis and computer‐simulated interventions, this study explored symptom‐level associations and potential intervention targets among nursing students.

**Methods:**

Participants enrolled in the “Be Resilient to Nursing Career” program completed the Mobile Phone Addiction Index, Perceived Stress Scale, and Morningness–Eveningness Questionnaire. Network analysis was conducted to identify central and bridge symptoms. Computer‐simulated intervention analysis was used to identify potential symptom targets within the network. Moderated network analysis was further performed to examine the moderating role of morningness–eveningness preference.

**Results:**

The central symptoms were inability to control craving (Str = 0.88, Bet = 2, Clo = 0.039, and EI = 0.77) and productivity loss (Str = 1.13, Bet = 3, Clo = 0.038, EI = 0.95). Feelings of nervousness emerged as an important bridge symptom between perceived stress and mobile phone addiction. Inability to control craving showed the strongest influence in the simulated intervention analysis and may represent a potential intervention target. Feelings of nervousness showed the greatest change in the simulated intervention analysis. Four significant three‐way interactions suggested a potential moderating role of morningness–eveningness preference.

**Conclusion:**

This study identified potential symptom targets associated with perceived stress and mobile phone addiction among nursing students through network analysis and computer‐simulated interventions. The findings may help inform future student support strategies and mental health management in nursing education. Morningness–eveningness preference may play a moderating role in the association between perceived stress and mobile phone addiction.

## 1. Introduction

According to a study from MyCOS Research, 79% of university students in China exhibit problematic mobile phone use in classroom settings [1, 2]. Mobile phone addiction (MPA) is a form of behavioral dependence characterized by uncontrolled and disproportionate smartphone engagement [3–5]. Extensive research has demonstrated significant associations between MPA and multiple adverse outcomes across physical, psychological, and social domains, including heightened stress perception, depressive symptoms, and sleep disturbances [6–10]. A relationship between perceived stress and MPA has been documented among nursing students [11]. However, evidence on the symptom‐level associations between perceived stress and MPA remains limited. Network analysis may help identify the complex symptom‐level associations between MPA and perceived stress.

Using mobile phones excessively as a way to relieve stress may not effectively reduce stress; instead, it may contribute to adverse psychological and behavioral outcomes, negatively affecting learning and daily life and potentially increasing the risk of depressive symptoms [12]. Thus, identifying potential symptom targets may help inform future priorities for student support and intervention. However, traditional network analysis treats symptom relationships as relatively static, which is unable to identify potential symptom targets [13]. Computer‐simulated interventions provide valuable perspectives on the complex dynamics and connections within symptom networks using advanced network analysis methods [14]. This technique evaluates how individual symptoms influence network dynamics and identifies symptoms with relatively greater influence within the network, providing insights into potential intervention priorities [15]. Therefore, computer‐simulated interventions may help identify potential symptom targets linking MPA and perceived stress.

Morningness–eveningness preference reflects individual differences in preferred sleep–wake timing over a 24‐h period and is associated with sleep–wake behavioral patterns [16]. Morningness preference involves waking up early and going to bed early, whereas eveningness preference favors waking up late and staying up late into the night or early morning [17]. Compared with morningness preference, eveningness preference is associated with a higher prevalence of adverse mental states such as stress [18, 19]. Recent research has discovered an inverse relationship between electronic media use and sleep duration, along with a positive link to sleep difficulties. Additionally, eveningness preference has been positively correlated with internet addiction [20, 21]. Furthermore, research has shown that a morningness preference is associated with better health behaviors, mental health, and sleep quality [22, 23]. Thus, morningness preference may be associated with the relationship between perceived stress and MPA. Therefore, morningness–eveningness preference may play a moderating role in the relationship between perceived stress and MPA. Moderated network analysis (MNA) can be used to examine how moderating variables influence the relationships between symptom nodes within networks [24]. In addition, findings from computer‐simulated interventions and MNA may provide insights into potential student support priorities and mental health management strategies in nursing education.

## 2. Theoretical Framework

According to allostatic load theory [25–27], when individuals are exposed to a stressful stimulus, they may enter a state of allostasis involving activation of the nervous system, which can be influenced by factors such as genetics, lifestyle, and social experiences. Stressful stimuli may be appraised as threatening experiences by individuals and may be accompanied by physiological and behavioral responses [28]. Perceived stress is considered a common stress‐related stimulus and may contribute to allostatic responses, which could be associated with maladaptive behaviors such as MPA [29].

However, not all individuals respond to perceived stressors in the same way. According to Lazarus’s theory of stress and coping [30], stress responses are influenced by individuals’ appraisal of stressors and their coping strategies. Factors such as personality traits and social support may influence this appraisal process [31, 32]. Therefore, some individuals may be less likely to perceive certain stimuli as threatening, which may reduce the likelihood of maladaptive coping behaviors such as MPA.

Notably, morningness–eveningness preference may represent a relevant moderating factor in stress appraisal and stress responses. Eveningness preference, which has been associated with circadian rhythm‐related physiological and behavioral characteristics, has also been linked to increased stress vulnerability [33–35]. Misalignment between biological rhythms and environmental demands may contribute to greater allostatic load and prolonged stress responses, potentially increasing the likelihood of perceiving frustration or discomfort as threatening experiences [36].

At the theoretical level, prolonged allostatic load may be associated with difficulties in emotional regulation and executive functioning [37, 38]. Individuals who perceive stress primarily as frustration or discomfort requiring immediate relief may be more likely to adopt impulsive coping strategies focused on short‐term rewards [39]. Such coping tendencies may be associated with a greater likelihood of impulsive‐compulsive behaviors, including MPA. Therefore, eveningness preference may be associated with a stronger relationship between perceived stress and MPA through stress‐related behavioral and coping vulnerabilities.

Based on these theoretical perspectives, the present study integrates the Allostatic Load Theory (focusing on cumulative physiological responses) with the cognitive appraisal perspective on stress.

We hypothesized that

**Hypothesis 1.**

Core and bridge symptoms would be recognized by the network analysis.

**Hypothesis 2.**

Potential symptom targets would be identified through computer‐simulated intervention analysis.

**Hypothesis 3.**

The moderation of morningness–eveningness preference between MPA and perceived stress would be recognized by MNA.

## 3. Methods

### 3.1. Design and Participants

A total of 439 nursing students were enrolled from “Be Resilient to Nursing Career (BRNC, [40–45])” across 4 universities in Guangdong, Shandong, Hunan, and Sichuan Provinces from October 2023 to December 2023. They were selected using convenience sampling. Inclusion and exclusion criteria have been described in past studies of this program [2, 40, 41]. Upon initial data inspection, it was observed that 13 questionnaires (3%) had some missing data. After these cases were excluded, a total of 426 valid questionnaires were retained (response rate = 97%).

## 4. Measures

### 4.1. Demographic Characteristics

Based on previous studies [2], we collected demographic characteristics including gender, grade, body mass index (BMI), residence, only child or not, and annual family income [40, 41, 44].

### 4.2. Measurement of MPA

The MPA Index (MPAI) invented by Chinese scholar Louis Leung [46] was used to assess MPA. The scale comprises 17 items covering four dimensions: inability to control cravings, feeling anxious and lost, withdrawal/escape, and productivity loss [47]. Each item is rated on a 5‐point Likert scale, ranging from 1 (“never”) to 5 (“always”) [48]. Higher overall ratings suggest more serious mobile phone use issues. Strong internal consistency was shown by the MPAI [49]. In this investigation, the Cronbach’s *α* value was 0.895.

### 4.3. Measurement of Perceived Stress

The Perceived Stress Scale (PSS) was used to evaluate the level of stress that respondents perceive in their lives [50]. The reliability and validity of the PSS have been well established in Chinese populations [51, 52]. The instrument consists of 14 items and comprises two subscales: feelings of uncontrollability and feelings of nervousness [53]. Each item is rated on a 5‐point Likert scale, ranging from 0 (“not at all”) to 4 (“always”), yielding a total score between 0 and 56 [53]. In this study, the Cronbach’s *α* value for the scale was 0.898.

### 4.4. The Morningness–Eveningness Questionnaire (MEQ)

The MEQ, devised by Horne and Östberg [54], was translated into Chinese by Li et al. [55]. This questionnaire serves as an instrument to assess whether an individual’s peak alertness occurs in the morning, evening, or during the intermediate period. It comprises 19 items on two dimensions including sleep phase and optimal sleep time [56]. Total scores range from 16 to 86 [57]. Higher scores indicate a stronger preference for morningness, whereas lower scores indicate a stronger preference for eveningness [54]. Based on the total score, participants are classified into five categories: Absolute Morning Type (70–86), Moderate Morning Type (59–69), Intermediate Type (42–58), Moderate Evening Type (31–41), and Absolute Evening Type (16–30) [54]. Cronbach’s *α* for domains ranged from 0.765 to 0.833 in the present study.

### 4.5. Data Analyses

The analysis began with computation of descriptive statistics.

Next, a network analysis was performed to explore the connections between MPA and perceived stress, aiming to identify the most central symptoms [58]. Network analysis enables the visualization of interactions between nodes within variables [59]. Notably, highly influential nodes may affect the states and behaviors of other nodes, so identifying them may help inform potential symptom management priorities [60]. Within symptom networks, bridge symptoms serve as connectors linking different symptom domains and may contribute to comorbidity between conditions [61, 62].The strength of bridge symptoms is considered an important indicator for identifying comorbidity patterns [63].

Subsequently, a computer‐simulated intervention was conducted to identify potential symptom‐specific intervention targets. The NodeIdentifyR algorithm, which utilizes binary data, was employed for this analysis [64]. Data were dichotomized based on the median: responses of “never” or “rarely” were coded as 0, whereas all other responses were coded as 1. Two types of simulated interventions were examined: alleviating interventions targeting low‐activation symptoms and aggravating interventions targeting highly activated symptoms [65].

Finally, the MNA approach was used to examine MEQ as a moderator in the pairwise interactions between two nodes. The mgm *R* package (version 1.2–14) was used to construct and visualize the MNA [24]. In MNA, variables are represented as nodes [66, 67]. To assess the stability of the estimated parameters, 1000 bootstrap samples were generated using the resample function in the mgm package [68].

In this study, we used SPSS 26.0, R4.4.1, and software packages such as “networktools”, “mgm”, “qgraph”, “bootnet”, and “nodeIdentifyR”.

### 4.6. Ethical Considerations

The research was approved by the Ethics Committee of the First Affiliated Hospital of Guangzhou University of Traditional Chinese Medicine, with the reference number (ZYYEC‐ERK【2020】132). Each subject granted informed consent after receiving a verbal description of the procedure and its objective, in compliance with the principles stated in the Declaration of Helsinki. The participants were given assurances regarding the confidentiality of their data and the preservation of their anonymity.

## 5. Results

### 5.1. Demographic Characteristics

The overall sample comprised 426 nursing students. Their average age was 19.23 (SD = 0.88). The majority of participants (333, 78.2%) were not only children. Only 18.1% of the participants were male, and 212 (49.8%) lived in urban areas. Additional demographic information is provided in Table 1.

**TABLE 1:** Demographic analysis among nursing students.

Variables	n (%)
Age (M ± SD)	19.23 ± 0.88
BMI (M ± SD)	20.32 ± 2.62
Grade	
1	62 (14.6%)
2	144 (33.8%)
3	112 (26.3%)
4	108 (25.4%)
Sex	
Male	77 (18.1%)
Female	349 (81.9%)
Residence	
City	212 (49.8%)
Countryside	214 (50.2%)
Only child	
Yes	93 (21.8%)
No	333 (78.2%)
Annual family income	
≤ 80000RMB	140 (32.9%)
80,000 ∼ 150000RMB	215 (50.5%)
≥ 150000BRMB	71 (16.7%)

### 5.2. Network Analysis of Perceived Stress and MPA

**FIGURE 1:**
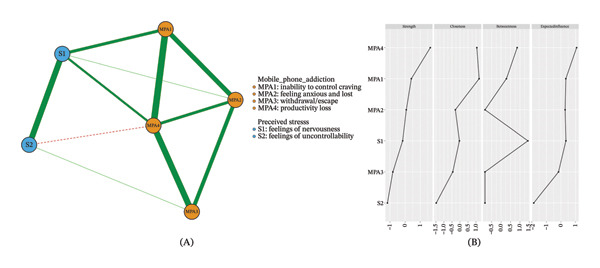
Network analysis visualization and centrality metrics for MPA and perceived stress.

The majority of nodes were positively correlated with one another. Strong correlations were observed between MPA4 and MPA1 (*r* = 0.35), MPA4 and MPA3 (*r* = 0.33), feelings of nervousness (S1) and feelings of uncontrollability (S2, *r* = 0.32), and MPA1 and MPA2 (*r* = 0.27). Between the two domains, a relatively strong correlation was identified between S1 and MPA1 (*r* = 0.21), whereas S2 showed a weak negative correlation with MPA4 (*r* = −0.08). In addition, MPA4 demonstrated the highest centrality indices (Str = 1.13, Bet = 3, Clo = 0.038, EI = 0.95), followed by MPA1 (Str = 0.88, Bet = 2, Clo = 0.039, EI = 0.77), indicating that these nodes were the most central symptoms in the network. The complete network model, including all relevant associations, is presented in Figure 1.

**FIGURE 2:**
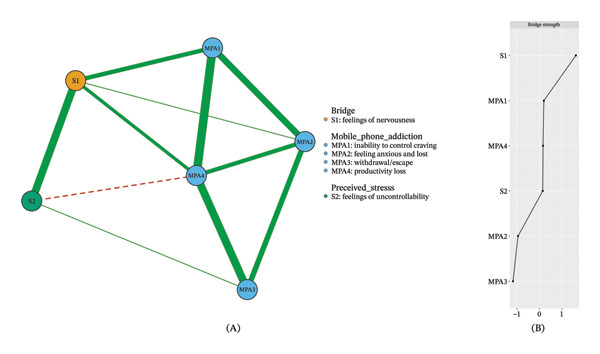
Bridge network analysis visualization and centrality metrics for MPA and perceived stress.

A bridge network analysis between perceived stress and MPA was conducted to identify bridge symptoms (Figure 2A). Node strength was used as a centrality metric to estimate comorbidity. The results showed that S1 demonstrated the highest bridge strength, indicating that it was the most influential bridge symptom in the network. The remaining node strengths are shown in Figure 2B.

### 5.3. Computer‐Simulated Interventions

**FIGURE 3:**
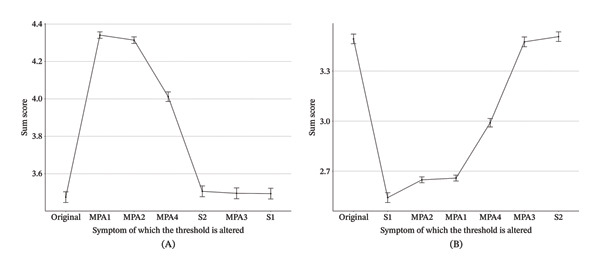
The effect of computer‐simulated interventions on the symptoms. (A) Estimated network changes in the simulated aggravating intervention analysis. (B) Estimated network changes in the simulated alleviating intervention analysis.

The outcomes of the computer‐simulated intervention analysis are displayed in Figure 3. In the simulated alleviating intervention analysis, S1 showed the lowest total score, suggesting that it may represent a potential symptom target associated with simulated symptom alleviation. In the simulated aggravating intervention analysis, MPA1 demonstrated the highest sum score and may represent a symptom with relatively greater influence within the network.

### 5.4. Moderated Network Analysis

**FIGURE 4:**
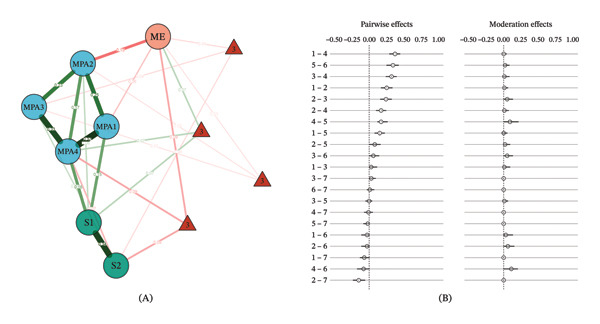
The moderated network model of MPA, perceived stress and morningness–eveningness preference. (A) Graph of the moderated network model. (B) Stability of the moderated network structure. MPA1 = inability to control craving; MPA2 = feeling anxious and lost; MPA3 = withdrawal/escape; MPA4 = productivity loss; S1 = feelings of nervousness; S2 = feelings of uncontrollability; ME = morningness–eveningness preference.

A moderated network model including MEQ was constructed and visualized (Figure 4A). Four significant three‐way interactions were identified that were related to MPA and perceived stress. Three interactions were negative. Higher MEQ scores indicate a greater preference for morningness, suggesting that morningness preference may weaken the associations between MPA3 and S2 (weight = 0.04), MPA4 and S2 (weight = 0.09), and MPA2 and MPA3 (weight = 0.03). However, one interaction showed a positive association, indicating that higher MEQ scores were associated with a stronger association between MPA4 and S1. Specifically, for every 1‐unit increase in MEQ, the correlation between MPA4 and S1 increased by 0.07. The stability of the moderated network structure is shown in Figure 4B.

## 6. Discussion

This study represents a novel attempt to integrate computer‐simulated interventions with traditional symptom network analysis to explore potential intervention priorities for psychological problems among nursing students. The simulation results suggest that “inability to control craving” may represent a potential target for preventive support strategies, whereas “feelings of nervousness” may be associated with broader changes across the symptom network. This “simulation‐based exploration” research framework extends beyond the static description of traditional network analysis by estimating the potential system‐level influence of different symptom targets prior to actual intervention implementation. As such, it may provide preliminary evidence to support the development of targeted student support and mental health management strategies in nursing education.

First, the central symptoms were inability to control craving and productivity loss. These findings suggest that feelings of nervousness were strongly associated with multiple MPA symptoms, which is consistent with previous studies [3, 69, 70]. In addition, feelings of nervousness demonstrated the strongest bridge centrality, suggesting that it may represent an important bridge symptom linking perceived stress and MPA. Previous research has suggested that individuals experiencing negative psychological states, such as anxiety, may engage in excessive mobile phone use to seek psychological reassurance [71]. While most nodes were positively correlated, a weak negative correlation was observed between feelings of uncontrollability and productivity loss. Past research has shown that lower perceived stress may be associated with higher productivity [72]. The present findings suggest that individuals experiencing stronger feelings of uncontrollability reported relatively lower productivity loss in this sample.

Second, the computer‐simulated interventions identified the inability to control cravings as a potential symptom target within the network. This result was consistent with the central symptoms confirmed by network analysis, suggesting that inability to control craving may demonstrate relatively greater influence within the symptom network [73]. Therefore, nursing educators and student support systems may pay additional attention to nursing students exhibiting difficulties controlling mobile phone cravings. Besides, the simulated alleviating intervention analysis suggested that feelings of nervousness may represent another potentially influential symptom target. Feelings of nervousness also demonstrated the highest bridge centrality within the network. Previous studies have shown that individuals experiencing nervousness or anxiety may use mobile phones as a coping strategy for negative emotions [71]. The more nervous an individual becomes, the harder it is for them to resist using their mobile phone, as it serves as a source of psychological comfort, much like a pacifier [74–76].

Third, morningness–eveningness preference moderated the relationship between perceived stress and MPA. According to the MNA results, higher morningness preference scores were associated with weaker relationships between withdrawal/escape and feelings of uncontrollability, productivity loss and feelings of uncontrollability, and feeling anxious and lost and withdrawal/escape. Nimrod’s findings suggest that individuals with a morningness preference may be more likely to use conventional media, whereas those with an eveningness preference may show greater preference for newer forms of media use [77]. Together with the simulated aggravating intervention analysis findings, morningness preference may represent a potentially relevant factor when considering preventive student support strategies.

Notably, because higher MEQ scores indicate greater morningness preference, this pattern may suggest relatively lower levels of nervousness and productivity loss among individuals with eveningness preference in this sample. However, it should be noted that the MEQ assesses sleep–wake preference rather than objective circadian rhythms; therefore, these findings should not be interpreted as evidence of physiological circadian or hormonal mechanisms. Although previous studies have reported associations between eveningness and alterations in the circadian regulation of stress‐responsive systems, including the hypothalamic–pituitary–adrenal axis, which may be related to perceived stress and coping behaviors such as mobile phone use [78–80], such mechanisms cannot be directly inferred from the present data. Therefore, the relationship between morningness–eveningness preference, perceived stress, and MPA requires further longitudinal and physiological investigation.

## 7. Limitations

This study has several limitations. First, although network analysis is an innovative approach, the associations between perceived stress and MPA identified in this study are primarily correlational and should not be interpreted as causal relationships. Such relationships have been widely documented in the existing literature, including both cross‐sectional and longitudinal studies conducted in student populations [8, 81, 82]. Second, the computer‐simulated intervention algorithm was based on the ferromagnetic Ising model. This model simplifies symptoms into binary categories, which may not fully capture the complexity of symptom interactions. Third, this study did not implement actual interventions targeting morningness–eveningness preference. Therefore, the potential implications suggested by the simulated intervention and MNAs require further validation through longitudinal and intervention studies.

## 8. Conclusion

This study identified core and bridge symptoms within the network of perceived stress and MPA among nursing students. Computer‐simulated intervention analysis suggested that certain symptoms, particularly inability to control craving and feelings of nervousness, may represent potential intervention priorities within the network. In addition, morningness–eveningness preference may play a moderating role in the association between perceived stress and MPA. These findings may help inform future student support systems and mental health management strategies in nursing education.

## Author Contributions

Hongman Li: conceptualization, data curation, methodology, software, and writing–original draft. Yingting Jiang and Zhenrong Shen: investigation, resources, and validation. Ying Xiong and Yihao Zeng: investigation and resources. Qihan Zhang and Qiaoling Chen: data curation. Zengjie Ye and Jiagen Xiang: supervision and writing–review and editing.

## Funding

This research was funded by the Cultivation of Guangdong College Students’ Scientific and Technological Innovation (No. pdjh2023b0131) and Guangzhou University of Chinese Medicine (GZUCM[2024]/No.278‐‐53, GZUCM[2023]/No.276‐‐57, GZY2025GB0924).

## Disclosure

All authors read and approved the final manuscript.

## Conflicts of Interest

The authors declare no conflicts of interest.

## Data Availability

The data that support the findings of this study are available from the corresponding author upon reasonable request.
